# Geroprotective insights into the natural metabolite spermidine in aging and age-related diseases

**DOI:** 10.1038/s41514-026-00448-9

**Published:** 2026-07-20

**Authors:** Zhirui Jiang, Sen Tong, James L. Kirkland, Yu Sun

**Affiliations:** 1 Center for Single-Cell Omics, School of Public Health, Shanghai Jiao Tong University School of Medicine, Shanghai, China; 2 CAS Key Laboratory of Tissue Microenvironment and Tumor, Shanghai Institute of Nutrition and Health, Chinese Academy of Sciences, Shanghai, China; 3 Center for Advanced Gerotherapeutics, Cedars-Sinai Medical Center, Pacific Design Center, West Hollywood, CA USA; 4 Division of Endocrinology, Diabetes and Metabolism, Cedars-Sinai Medical Center, Los Angeles, CA USA; 5 School of Pharmacy, Institute of Aging Medicine, Shandong Medical and pharmacological University, Yantai, China

**Keywords:** Biochemistry, Cell biology, Physiology

## Abstract

Aging is associated with increased oxidative stress, which leads to pathological vulnerability. Spermidine is a ubiquitous natural polyamine found across all living organisms studied so far and present in multiple food sources. In humans, spermidine levels decline with aging, and a possible connection between reduced endogenous spermidine concentration and age-related organ degeneration has been proposed. We outline recent studies that address the potential contribution of spermidine to geroprotection, with a perspective of advancing spermidine towards clinical trials.

## Introduction

Aging is an inevitable process that gradually affects physical and mental capacity, including functional decline of vital organs. The number of people aged 65 years or over is projected to reach 1.6 billion by 2050, *per* the United Nations’ World Population Prospects of 2022^1^. With the expansion of the aging population, physical and mental dysfunction has become an increasingly pressing challenge. Safe, efficient and durable interventions to prevent, delay or slow declines in organ function in elderly populations are urgently needed. Polyamines, including spermine, spermidine and putrescine, are organic cations that play key roles in regulating cell proliferation, differentiation, survival and autophagy. More precisely, putrescine can be classified as a diamine, rather than a polyamine. Among these polyamines, spermidine is present in daily human nutrition, and can generate prominent cardioprotective and neuroprotective effects in rodent models^2^. Being naturally found in various dietary sources, spermidine has anti-inflammatory, antioxidant, and anticancer potentials^3^. External supply of spermidine can extend lifespan in model organisms including yeast, nematodes, flies and mice^4^. A study reported that spermidine supplementation with a spermidine-rich plant extract is safe and well-tolerated in both mice and older adults, prompting longer-term intervention in humans to investigate the impact of spermidine treatment^5^.

Recognized as an inducer of autophagy, spermidine is considered a geroprotective compound with properties resembling those of caloric restriction, positioning it as a potential “caloric restriction mimetic”^6^. Several independent clinical studies demonstrated that acute nutrient deprivation (fasting) causes an elevation in spermidine biosynthesis in organisms including yeasts, flies, mice and humans^7–10^. Moreover, spermidine restores autophagic flux in circulating lymphocytes of aged humans^10,11^, while increased dietary spermidine uptake is linked with reduced overall mortality in human populations^12^. Conversely, genetic or pharmacological blockade of spermidine increase *via* inhibition of ornithine decarboxylase 1 (ODC1) interferes with the pro-autophagic and possible geroprotective effects of fasting and rapamycin^13^. Perturbing the polyamine pathway in vivo abrogates the healthspan- and lifespan-extending effects, as well as the anti-arthritic and cardioprotective consequences of fasting^6^. These findings suggest spermidine as not only a “caloric restriction mimetic” with its supplementation mimicking the beneficial effects of nutrient deprivation but also a downstream effector of the possibly geroprotective effects of fasting and rapamycin. The proximal pharmacological target of spermidine is indeed the same as that of salicylic acid, the active metabolite or aspirin, both suppressing EP300 by competing for acetyl coenzyme binding^14^. To date, aspirin is probably the one single drug which generates the broadest positive impact on human mortality caused by cardiovascular and malignant disorders^15^. Although the literature on the longevity-enhancing effects of spermidine has been limited to model organisms, two prospective population-based studies report for the first time that spermidine uptake as a nutrient can reduce cardiovascular and cancer-related mortality in humans^12^.

Previous studies have suggested the potential of spermidine for regulating cell proliferation and growth, inhibiting inflammatory responses, modulating gene transcription and protein stability^2^. These functions suggest that spermidine may play a multifaceted role in supporting organismal health during aging. Here we review recent advances in understanding about the impact of spermidine on natural aging. Specifically, our current understanding of spermidine in treatment and prevention of organ dysfunction in the course of aging and with age-related diseases is improved. We propose consideration of moving toward clinical trials to test the potential impact of dietary supplementation with spermidine or spermidine-rich foods as a strategy for improving organismal health and preventing age-related pathologies. Notably, fortified foods have their benefits over supplementation, such as that they can merge into existing diets effortlessly and do not require humans to buy separate pills, a strategy distinct from simply administering spermidine supplements to volunteers.

## Biosynthesis and catabolism of spermidine in eukaryotes

Putrescine is the most abundant polyamine in the gut, while the fecal concentration of putrescine declines with aging as compared to young controls^16^. Since putrescine can be actively converted to spermidine and spermine, age-related changes in the gut microenvironment may impact bioactive polyamine production^17,18^. Commensal gut bacteria-derived spermidine can contribute to pathogenesis of several disorders, such as irritable bowel syndrome (IBS)^19^. Investigations using single-strain stable isotope-resolved metabolomic (SIRM) and in silico analyses identified important contributors to polyamine biosynthesis, *Bacteroides* spp., which harbor essential genes for this process with a potential to drive the upregulation of polyamines in inflammatory bowel disease (IBD)^20^. Specifically, *Bacteroides* spp. promote the biosynthesis of both putrescine and spermidine, with the arginine-agmatine-spermidine pathway predominating over the ornithine-putrescine-spermidine pathway in spermidine production within *Bacteroides*^20^. In the case of IBD, spermidine supplementation is neither necessary nor practical, as its levels are already increased as compared with healthy individuals. As spermidine can directly trigger visceral hypersensitivity, spermidine supplementation might pose a potential issue for healthy individuals, at least upon short term administration. However, the effect of longer term treatment with spermidine may need to be explored, particularly in terms of gastrointestinal sensitivity or discomfort. Dysregulated polyamine metabolites are also linked to the dysfunction of immune system and colon tumorigenesis^21^. In the glioblastoma microenvironment, spermidine is elevated and functions as a cancer cell-derived metabolite, which drives tumor progression by reducing the numbers and function of CD8^+^ T cells^22^. However, in colitis-associated carcinoma, *Apc* deletion-driven tumor development is reduced by spermidine^23^. The probiotics Bifidobacterium lactis (Bifal) LKM512 appears to prevent chronic inflammation of the colon and increase longevity by increasing concentrations of gut spermidine and spermine in aged mice^24^. A study investigating the impact of LKM512 yogurt on improvement of intestinal environment of the elderly indicated that polyamines increased by the administration of LKM512 yogurt can generate anti-inflammation and antimutagenicity effects in the intestinal tract^25^. Thus, spermidine’s biological effects are multifaceted and can vary depending on the disease types and pathological settings.

As the complex gut microbiome influences host aging and is linked with age-related disorders, restoring a healthy gut microbiome *via* fecal microbiota transplantation (FMT) holds the potential to therapeutically transfer healthy longevity^26^. Specifically, FMT from spermidine-treated mice can reduce the abundance of *Firmicutes* and increase the amount of *Bacteroidetes* and *Deferribacteres* in recipient mice^27^. Transplantation of spermidine-modified microbiota confers protection against obesity-increased colon length and reduces plasma lipopolysaccharide (LPS) levels, which are partially driven by a short-chain fatty acids (SCFA)-producing bacterium, the *Lachnospiraceae* NK4A136 group^27^. These findings suggest that spermidine supplementation may serve as a viable therapeutic solution for obesity.

**Fig. 1:**
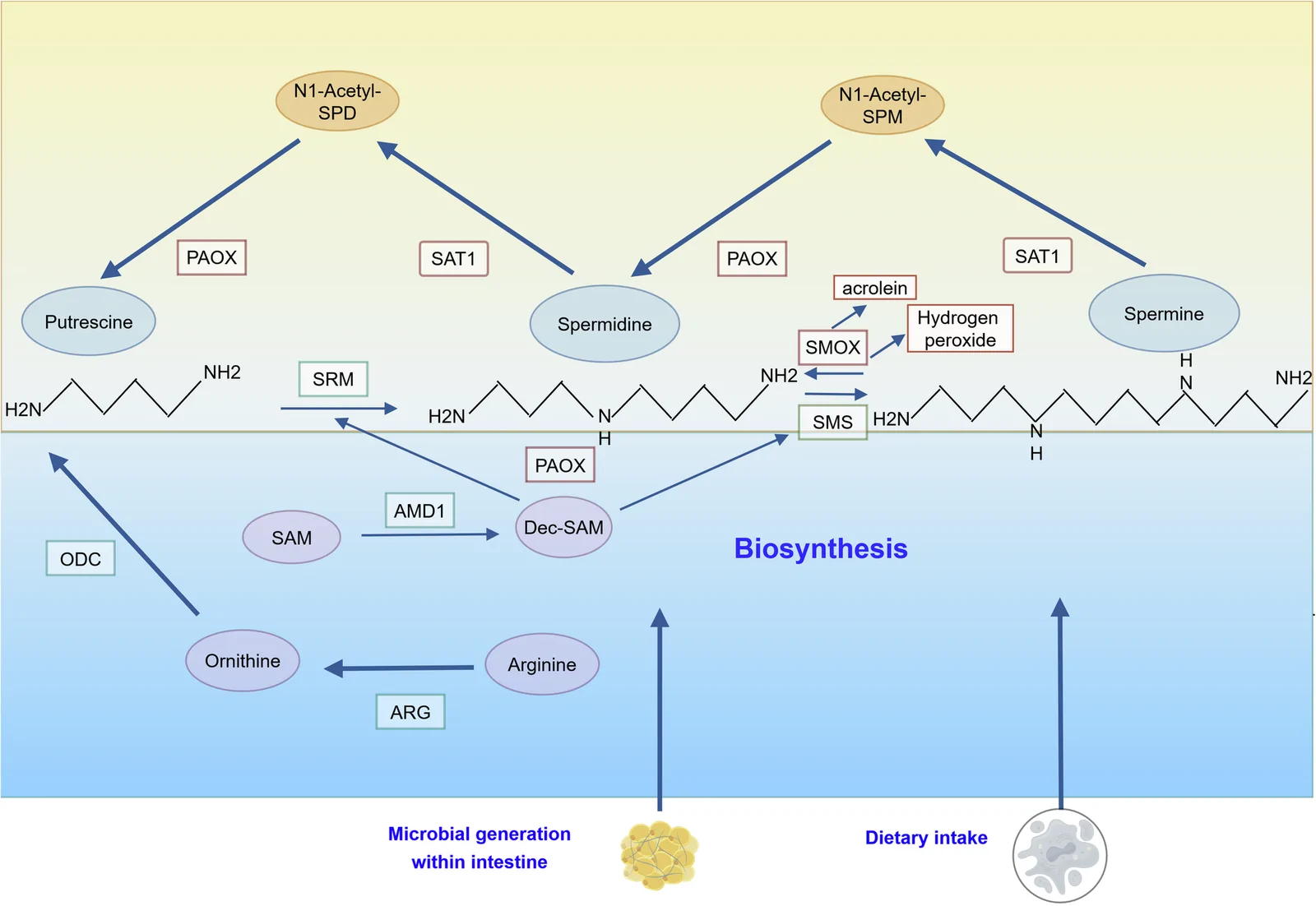
A landscape of polyamine metabolism. Intracellular polyamine is subject to a dynamic regulation of biosynthesis, catabolism, interconversion and transport. In the canonical biosynthetic pathway, ODC catalyzes the formation of putrescine from ornithine, as a rate-limiting and tightly regulated step. According to Uemura et al. (2026), the mechanisms are as follows: AMD1 generates decarboxylated *S*-adenosylmethionine, which provides aminopropyl groups required for conversion of putrescine to spermidine and spermidine to spermine. In contrast, polyamine degradation occurs mainly through two routes. Spermidine and spermine undergo *N*¹-acetylation by SAT1 before being subsequently oxidized by PAOX. Second, spermine is directly converted to spermidine by SMOX, yielding acrolein and hydrogen peroxide as byproducts. Although age-related declines in polyamines are well studied, the relative contributions of reduced biosynthesis, enhanced catabolism, and altered transport remain to be clarified^17^. ODC ornithine decarboxylase, AMD1 *S*-adenosylmethionine decarboxylase, SAM S-adenosylmethionine, De-SAM decarboxylated SAM, SMS spermine synthase, SRM spermidine synthase, SMOX spermine oxidase, SAT1 spermidine/spermine N^1^-acetyltransferase 1, PAOX polyamine oxidase. The figure is created with FigDraw.com.

Polyamines can be biosynthesized de novo in mammalian cells, and their metabolism is evolutionarily conserved across eukaryotes (Fig. 1). Arginine is linearly converted into ornithine, putrescine, spermine, and spermidine under the control of several enzymes including arginase (ARG), ornithine decarboxylase (ODC), spermine synthase (SMS) and spermidine synthase (SPDSY)^28^. Polyamine-blocking therapy, a strategy combining polyamine biosynthetic suppression with simultaneous inhibition of polyamine transport, is more effective than therapies based on polyamine depletion alone and may involve an anticancer immune response^28^. Clinical trials establishing the safety and dose level of AMXT-1501 (a polyamine transport inhibitor) dicaprate alone or in combination with DFMO (an ornithine decarboxylase inhibitor), and analyzing how AMXT-1501 and DFMO affect tumor metabolism are completed or ongoing (NCT03536728 and NCT05717153, respectively). Oral arginine administration increases putrescine levels in the colon and spermidine and spermine in the blood of mice^29^. Animals fed arginine in combination with the probiotic *bifidobacteria* LKM512 in a long-term study indicated attenuated inflammation, improved health and protection against age-related memory impairment, suggesting that intake of arginine and LKM512 may prevent age-dependent declines *via* polyamine upregulation^24^.

Spermine oxidase (SMOX) converts spermine back into spermidine through oxidative degradation. Spermidine/spermine N^1^-acetyltransferase 1 (SAT1) and polyamine oxidase (PAOX) are involved in acetyl-CoA-dependent acetylation and subsequent oxidation of spermine and spermidine, facilitating the reverse conversion of spermine and spermidine into putrescine^2^. Mice with Smox- and Sat1-deficiency can have elevated polyamine levels and aggregated α-Synuclein in the cerebellum, cerebellar damage, and severe ataxia^30^, indicating that lack of enzymes associated with spermidine catabolism may cause polyamine overproduction and lead to brain dysfunction. This is in contrast to health-protective and lifespan-extending effects of dietary spermidine, which are correlated with a reduced risk for cognitive impairment in humans^31^, a phenomenon observed in natural conditions.

## Altered spermidine levels in older subjects, including those with neurodegenerative conditions

Polyamine levels in the serum and urinary of humans progressively decline with increasing age and there is a significant negative correlation between serum spermidine concentration and organismal age^32,33^. Former studies disclosed an age-dependent decline of spermidine in human blood^33–35^. Relevant data suggest that nona/centenarians have a significantly higher circulating level of spermine with respect to total polyamines, as compared to the other age groups^34^. Spermidine concentration decreases with age in peripheral blood mononuclear cells (PBMCs) and red blood cells (RBCs)^10,11,36^. An interventional study focusing on polyamine-rich diet reported that spermine-induced lymphocyte function-associated antigen-1 (LFA-1) suppression is associated with the inhibition of aberrant gene methylation, and dietary polyamine contributes to both the health and longevity of humans^37^.

According to Madeo et al.^2^, however, polyamine levels can increase in some age-related pathologies, including stroke, chronic renal failure, heart failure, chronic atypical neutrophilic dermatosis and neurodegenerative conditions including mild cognitive impairment (MCI) and Alzheimer’s disease (AD)^38–42^. Although these changes may be attributable to adaptive responses of cells and/or tissues to exogenous stress, blood-based polyamine profiling of patients is likely a feasible strategy to improve disease diagnosis and optimize patient stratification. An increase in protein-conjugated acrolein (PC-Acro) in plasma can serve as a good biochemical marker in patients with stroke or chronic renal failure^38^. Technically, high-resolution mass spectrometry can be combined with high-performance liquid chromatography to measure serum levels of polyamines and identify possible diagnostic circulating biomarkers, potentiating a more accurate evaluation of lymphoma patient diagnostic stratification and robust comparisons between patient groups^43^.

Given the involvement and importance of spermidine in the maintenance of various cellular functions, dysregulation of spermidine may contribute to the pathogenesis of multiple diseases. An epidemiological study identified a non-linear correlation between spermidine concentration in serum and MCI prevalence, with a higher serum spermidine level potentially linked with a lower risk of MCI events^44^. Spermidine levels in serum tend to decrease in patients with MCI or AD as compared to those of healthy controls^45^. Notably, a recent study demonstrated that spermine effectively extends lifespan, alleviates movement deficits, as well as restores mitochondrial function in *Caenorhabditis elegans* engineered to express Alzheimer’s Tau and Parkinson’s α-synuclein^46^. This finding unveils the mutual interplay between spermine, protein condensation and functional outcomes, advancing our understanding of these neurodegenerative diseases and provides a baseline for alternative and novel therapeutic development. Although the data mainly derived from worms, future efforts are warranted to explore the effects of spermidine in humans and to determine if there are species-dependent consequences.

Although increased plasma spermidine levels are linked to better AD scores in subjects^47^, they can paradoxically indicate elevated risks of cognitive impairment among patients with ischemic stroke in the poststroke stage^48^. As higher plasma polyamine levels are associated with increased risk of poststroke cognitive impairment (PSCI) among patients with ischemic stroke, plasma polyamines may be implicated in the pathophysiologic processes of PSCI and represent potential intervention targets for PSCI. Indeed, the increase of spermidine level may be a compensatory response during disease progression or a consequence of changes in metabolic enzymes^49^. Specifically, in the ornithine metabolism pathway, a decreased concentration of SMS, which catalyzes spermidine conversion to spermine, was detected in AD subjects when compared to healthy controls in a Nakayama Study conducted in Iyo City, Ehime Prefecture^50^. Loss-of-function mutations of SMS cause Snyder-Robinson Syndrome (SRS), a recessive X-linked syndrome characterized by intellectual disability and several other conditions. Beyond decreased spermine and increased spermidine in SRS cells, ODC activity and its product putrescine were significantly reduced^51^. Knockdown of ODC, SRM and SMOX reduced α-synuclein toxicity, while inhibition of SAT1 and SMS exacerbated the toxicity, underscoring the critical role of polyamine pathways in regulating the effect of α-synuclein and offering novel therapeutic targets for Parkinson’s Disease (PD)^52^.

Both observational and interventional studies have recently been conducted to determine the relevance of spermidine for cognitive aging. Observational studies have investigated the association between spermidine levels and aging-related cognitive conditions, yielding mixed results^53^. Several studies suggest a potential link between higher spermidine levels and optimized cognitive performance. For instance, dietary spermidine intake is correlated with a reduced risk of cognitive impairment, increased hippocampal volume and elevated cortical thickness^54^. For interventional studies, spermidine supplementation may represent a strategy to mitigate memory decline in older adults. Three out the 4 interventional trials reported positive effects of spermidine supplementation on cognitive function, such as improvements in memory performance and augments in cognitive capacity^55–57^. However, in a randomized clinical trial, longer term supplementation of spermidine in participants with subjective cognitive decline failed to modify memory and biomarkers, with possible beneficial effects on both inflammation and verbal memory to be validated in future at higher dosages^58^.

While we cannot exclude the potential impacts of pathological severity and disease subtypes, contradictory data in the serum, urinary, tissue or organ levels of spermidine between patients manifesting cognitive decline and healthy controls may be due to sample sizes or detection methods employed. Moreover, spermidine may have beneficial effects, which can be attributed to its metabolites, since spermidine supplementation significantly enhances plasma spermine levels in healthy adults without affecting the concentration of spermidine per se or putrescine^59^. Thus, dietary spermidine can be pre-systemically converted into spermine, with the in vitro and clinical effects of spermidine being at least partially attributable to its anabolic metabolite, spermine.

## Dysregulated spermidine metabolism is associated with age-related disorders

As amyloid beta 1-42 (Aβ_1–42_) hastens caspase-dependent apoptosis, reinforces neuronal cell death and enhances intracellular polyamine concentrations in cultured neuronal cells^60,61^, it is possible that amyloid peptide deposition in AD brains may disturb spermidine metabolism. In cultured hippocampal neurons, treatment with Aβ leads to elevated activity of ODC, a critical rate-limiting enzyme in polyamine biosynthesis, as well as spermidine uptake^62^.

While polyamine synthesis following a short-term stressor appears to be beneficial, persistent stress and subsequent polyamine stress responses may cause maladaptive polyamine dysregulation and thereby contribute to neuropathology and AD progression. A study examining the effects of chronic intermittent restraint stress on polyamine concentrations in adult male C57BL/6 mouse hippocampus indicated that chronic treatment with α-difluoromethylornithine can reduce brain polyamine concentration and modulate emotional response to novelty stress^63^. Moreover, mice with the tau P301L mutation (rTg4510) exhibit an increased level of spermidine acetylation in the hippocampus, with elevated tau fibrillization and oligomerization^64^. Thus, there is a novel link between tau pathology and spermidine dysfunction, while targeting specific arms within the polyamine pathway may alleviate certain features of the tau phenotype^64^.

ATPase cation transporting 13A2 (ATP13A2) facilitates cellular uptake of polyamines such as spermidine by endocytosis and transports them into the cytosol, underscoring the role for endolysosomes in the uptake of polyamines into cells^65^. Defective lysosomal polyamine export was proposed to be a mechanism for lysosome-dependent cell death, which may be implicated in neurodegeneration, highlighting the importance of the mammalian polyamine transport system^65^. On the other hand, earlier studies suggest that spermidine promotes the aggregation of α-synuclein, a critical pathological hallmark of dementia with Lewy bodies and Parkinson’s disease (PD), while spermine increases the cellular toxicity of α-synuclein, suggesting the possibility that dysregulated polyamine homeostasis may accelerate pathological progression in neurodegenerative diseases (also briefly discussed earlier)^66–68^. However, whether spermidine level disturbance is a cause or consequence of these age-related pathological conditions remains unclear.

Sepsis-induced cardiomyopathy (SICM) is a disease characterized by impaired mitophagic flux, mitochondrial dysfunction and oxidative stress. Spermidine can mitigate SICM by restoring mitochondrial quality control, normalizing mitophagic flux and stabilizing cellular redox homeostasis^69^. Spermidine supplementation can counteract α-defensin expression and dysbiosis of the microbiome, phenotypes caused by loss of SMOX, suggesting its potential as an adjunctive treatment for colitis and a chemopreventive agent for colon carcinogenesis^23^.

A study examining spermidine in the links between autophagy and systemic metabolism found that spermidine generates minimal metabolic effects in young and old mice under normal nutrition conditions^70^. However, spermidine counteracts high-fat diet (HFD)-induced obesity by increasing lipolysis in visceral fat, mechanistically through upregulating expression of the hepatokine fibroblast growth factor 21 (FGF21) in liver without reducing food intake^70^. Another study demonstrated a decrease in spermidine and activation of RIPK1 in the vascular tissues of human patients developing diabetes, and reveals that spermidine mediates acetylhypusination of RIPK1 to prevent diabetes onset and progression^71^.

Sarcopenia is a pathological condition characterized with gradual loss of muscle mass, muscle strength and physical performance. Spermidine levels are higher and the spermine/spermidine ratio is lower in the sarcopenia group than in the healthy group^72^. Similarly, whole-blood spermidine levels are higher and spermine/spermidine ratio is lower in patients undergoing hemodialysis, an approach that compensates for polyamine excretion in patients with renal failure^73^. These studies suggest that the blood spermine/spermidine ratio represents a candidate diagnostic indicator for the risks of sarcopenia and renal dysfunction.

Nutritional interventions including calorie restriction (CR) and rapamycin extend healthspan *via* molecular mechanisms converging on suppression of the mechanistic target of rapamycin complex 1 (mTORC1)^74,75^, a molecule that antagonizes autophagy induction^76,77^. Spermidine, which acts as a downstream effector of fasting (nutrient deprivation) and rapamycin (a known caloric restriction mimetic)^13^, differs in its effects from these pathways, mainly by promoting autophagy, a cytoplasmic recycling mechanism, which will be discussed below.

## Spermidine restores physiological function by activating autophagy

Autophagy is a fundamental and critical cellular activity that maintains the functional homeostasis of intracellular proteins and turnover of diverse organelles. Dysfunctional autophagy is linked to an array of age-related disorders^78,79^. A study involving hippocampal injections of genetic and pharmacological regulators of autophagy suggests that autophagic induction in hippocampal neurons is necessary to form novel memories by increasing activity-dependent synaptic plasticity, such as dendritic spine formation and neuronal facilitation as well as long-term potentiation^80^. According to this study, systemic administration of young plasma, which contains the bone-derived molecule osteocalcin, into aged mice can rejuvenate memory in an autophagy-dependent manner, supporting a role for autophagy in mediating communication between systemic factors and neurons to facilitate cognition^80^. In the context of neurodegeneration, the primary role of spermidine is to clear misfolded proteins by serving as a conserved apoptotic metabolite messenger^81^. Moreover, genetic or pharmacological activation of autophagy is sufficient to extend lifespan in model organisms, including flies and mice^82^. Of note, advanced age is associated decreased autophagy and increased cognitive impairments^83^. Thus, despite reduced hippocampal autophagic activity during aging, restoring autophagy can at least partly reverse age-related memory loss, implying a potential therapeutic benefit of modulating autophagy in aged brains to prevent age-related cognitive impairment.

**Fig. 2:**
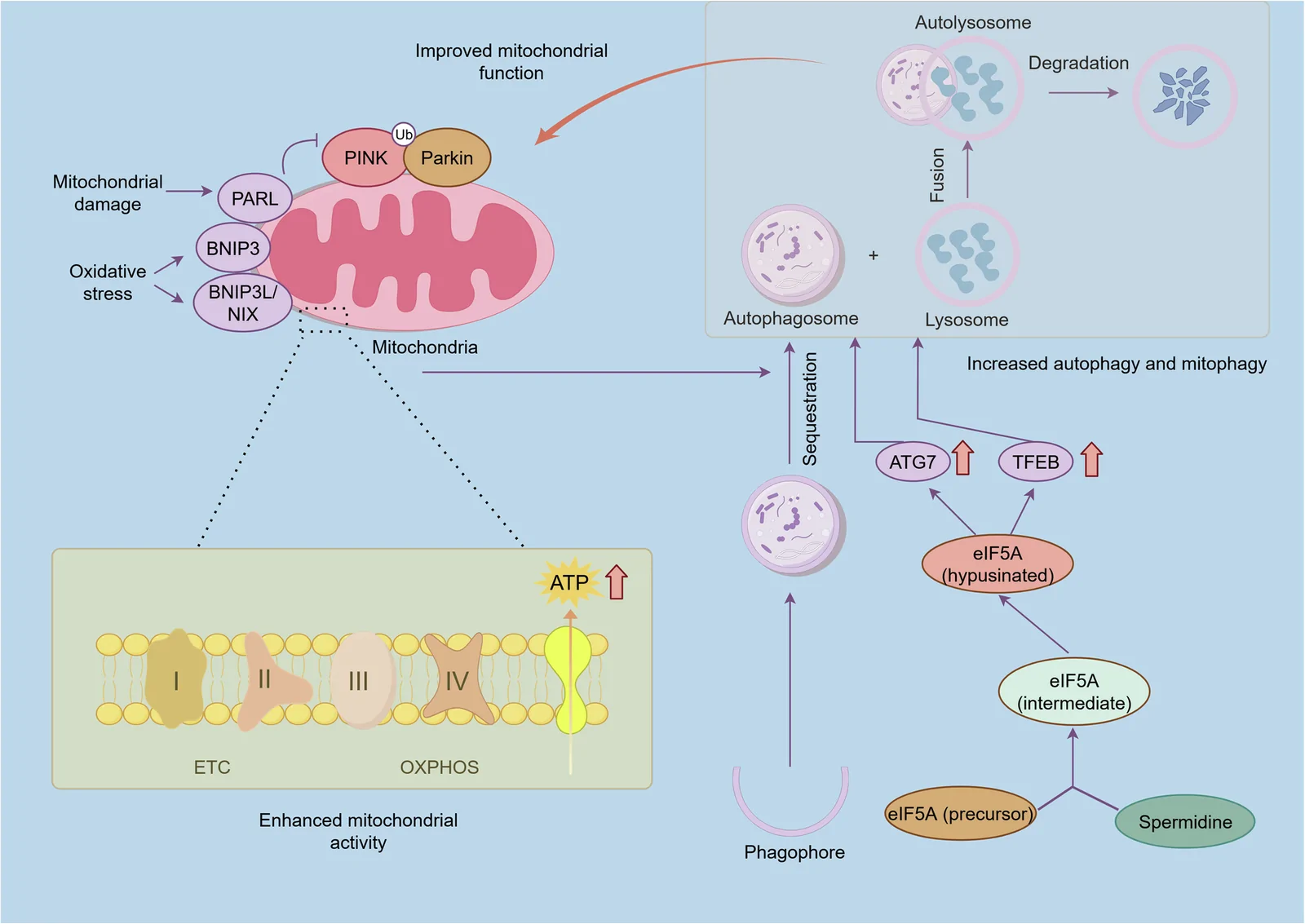
Spermidine provokes autophagy, enhances mitophagy and improves mitochondrial function. Spermidine preserves autophagy by hypusinating eIF5A, which promotes the synthesis of TFEB and ATG7^160^, the latter two factors subsequently enhancing autophagy activity in eukaryotic cells. Spermidine also regulates mitochondrial quality control under pathophysiological conditions *via* multiple pathways, including mitochondrial mass maintenance, proteostasis enhancement and improvement of mitochondrial function. Mitochondrial damage activates intramembrane protease PARL, while BNIP3 and BNIP3L/NIX^161^, paralogous membrane proteins on the outer mitochondrial membrane, serve as ubiquitin-independent mitophagy receptors in response to oxidative stress. PINK1 and the cytosolic E3 ubiquitin ligase Parkin are key regulators of mitophagy^162^. Dietary spermidine intake can improve mitochondrial function in aged organisms depending on the mitophagy regulators PINK1 and Parkin as well as the autophagy regulator ATG7. eIF5A, eukaryotic translation initiation factor 5A. TFEB transcription factor EB, ATG7 autophagy-related gene 7, PARL presenilin associated rhomboid like, BNIP3 BCL2 interacting protein 3, BNIP3L/NIX BCL2 interacting protein 3 like, PINK1 PTEN-induced kinase-1. The figure is created with FigDraw.com.

NaHS-mediated activation of the hippocampal ODC/spermidine pathway improves autophagic flux and counteracts cognitive decline in rats with streptozotocin (STZ)-induced diabetes^84^. This is evidenced by the reduced number of autophagosomes and downregulated expression of Beclin-1, LC3-II and p62 in the hippocampus of rats co-treated by NaHS and STZ. Treatment with spermidine and TAT-Beclin 1, an autophagy agonist, rescues memory capacity and GluA1 post-translational modifications by promoting autophagy-mediated degradation of amyloid fibrils, supporting the notion that autophagy enhancers can promote the degradation of misfolded proteins to attenuate age-related memory decline^85^. In young male rats induced to develop a premature aging-like state by D-galactose, spermidine protects against oxidative stress by regulating autophagy and antioxidant levels, enhances the activities of electron transport chain (ETC) complexes in mitochondria and minimizes neuroinflammation, suggesting beneficial effects of spermidine during aging and with chronic diseases^86^. As a substrate of hypusination (will be discussed below), spermidine induces autophagy in vivo and rejuvenates memory B cell responses to prevent immune senescence, by post-translationally modifying the translation factor eIF5A, a molecule essential for synthesis of the autophagic transcription factor EB (TFEB)^10^ (Fig. 2).

In 5×FAD mice, apoptotic neurons or glial cells actively release spermidine, which functions in a paracrine manner to induce autophagy in neighboring cells, promoting cargo encapsulation within autophagosomes and subsequent degradation in autolysosomes in nearby cells^81^. Thus, there is a link between cellular apoptosis and autophagy in the case of AD, for which spermidine supplementation may be an effective therapeutic strategy. As regard to the inflammatory microenvironment, treatment with exogenous spermidine increases osteogenesis of bone marrow mesenchymal stem cells (BMSCs) in an inflammatory setting and enhances N6-methyladenosine (m^6^A) methylation, which plays a significant role in the regulation of gene expression^87^. In this case, spermidine activates autophagy, while S-adenosylhomocysteine (SAH) inhibits spermidine-induced autophagy, indicating that spermidine augments the osteogenic potential of BMSCs *via* an m^6^A methylation-autophagy axis under inflammatory conditions^87^. Alternatively, in ɑ-Klotho-deficient mice, a model of accelerated aging, spermidine treatment increased the number of mitochondria and suppressed mitophagy of primary salivary gland cells (PSGCs), as indicated by downregulation of BNIP3 and LC3B^88^. Thus, mice in an accelerated aging-like state have disturbed mitochondrial homeostasis and dysfunctional autophagy/mitophagy pathways in PSGCs, likely *via* dysregulated spermidine metabolism^88^.

A study involving oral supplementation with polyamines suggested that oral spermidine administration can enhance cardiac autophagy and mitophagy as well as mitochondrial respiration^9^. Furthermore, spermidine treatment improved the mechano-elastic properties of cardiomyocytes in vivo, augmented titin phosphorylation and protected against cardiac hypertrophy and diastolic dysfunction, which together delayed progression to heart failure in mice.

In diabetic animals spermidine treatment can mitigate severe hyperglycemia, follicular and endometrial degeneration, fibrosis and antioxidant enzymes glutathione (GSH), superoxide dismutase (SOD), catalase (CAT) and glutathione peroxidase (GPx), as well as nuclear factor erythroid 2-related factor 2 (Nrf2) depletion, through upregulation of antioxidant defense factors and downregulation of inflammatory inducers including malondialdehyde (MDA) and transforming growth factor-beta (TGF-β)^89^. In contrast to Urolithin A, an ellagitannin-derived natural metabolite, which stimulates mitophagy through pathways including PINK1/Parkin RBR E3 ubiquitin protein ligase, spermidine induces autophagy by regulating critical signaling pathways such as 5′ AMP-activated protein kinase (AMPK) and sirtuin 1 (SIRT1), hence mitigating age-related cellular dysfunction and promoting cellular homeostasis^90^.

## Spermidine regulates acetylation of intracellular proteins

Acetylation of cellular and nuclear proteins is an important epigenetic regulatory mechanism^91^. Spermidine activates autophagy *via* suppression of acetyltransferases and stimulation of deacetylases, together shaping an autophagy-active state^92,93^. Polyamine catabolism usually involves acetyl-CoA consumption, a process that is coupled to protein deacetylation, while disturbed spermidine synthesis and decreased eIF5A hypusination may enhance acetyl-CoA biogeneration *via* indirect mechanisms^94,95^.

In aging yeast, treatment with spermidine can trigger epigenetic deacetylation of histone H3 *via* inhibition of histone acetyltransferases (HATs), preventing oxidative stress and necrosis^8^. Conversely, depletion of endogenous polyamines results in hyperacetylation, generation of reactive oxygen species (ROS), early necrotic death and shortened lifespan. Spermidine acts on epigenetic enzymes such as E1A-binding protein p300, HAT enzymes including Iki3p and Sas3p, and α-tubulin acetyltransferase 1, which functionally modulates autophagy^96^.

Induction of autophagy by some natural agents such as resveratrol requires the nicotinamide adenine dinucleotide (NAD)-dependent deacetylase SIRT1. However, spermidine-mediated autophagy appears to be generally independent of SIRT1, at least in human and yeast cells as well as in nematodes^97^. While resveratrol and spermidine trigger autophagy *via* distinct mechanisms, these compounds engage convergent pathways, which culminate in concordant modifications of the acetylproteome^97^. Of note, a cytoplasm-restricted SIRT1 mutant can stimulate autophagy, implying that cytoplasmic deacetylation dictates the autophagic cascade^97^. A study investigating the effect of spermidine on vascular calcification disclosed that spermidine can induce SIRT1 upregulation in vascular smooth muscle cells (VSMCs) and endoplasmic reticulum (ER) stress signaling component downregulation^98^. To the contrary, pharmacological inhibition or knockdown of SIRT1 markedly suppressed the effect of spermidine on VSMC calcification, further substantiating that spermidine alleviates vascular calcification by upregulating SIRT1 and restraining ER stress^98^. It has been reported that spermidine administration results in upregulated expression of SIRT1, PGC-1α, NRF1, NRF2 and TFAM, reduced ROS production and improved OXPHOS performance in senescent cardiomyocytes^99^. The findings suggest that spermidine helps promote the antioxidant potential and reduce intracellular stress pressure.

Of note, autophagy was not analyzed in these studies, and whether the role of spermidine in modulating SIRT1 expression is linked with autophagy-related mechanisms remains unclear. Indeed, there may be synergy between spermidine-mediated inhibition of acetyltransferase activity and SIRT1 deacetylase activity. As SIRT1 activity can be inhibited through acetylation, spermidine-induced autophagy may be potentially driven by SIRT1 expression and its sustained deacetylase activity.

## Spermidine-mediated hypusination and its implications

Hypusine is a unique amino acid that is post-translationally synthesized on eukaryotic initiation factor 5A (eIF5A, a highly conserved and abundant protein comprising 157 amino acids) from spermidine by deoxyhypusine synthase (DHPS). Hypusination of eIF5A plays a key role in eukaryotic translation, including initiation, elongation and termination, despite its name^100^. Besides engagement in protein synthesis, this highly conserved modification activates eIF5A, subsequently promoting autophagy by driving the translation of the transcription factor TFEB and autophagy-related proteins. The spermidine-hypusinatio-TFEB-autophagy axis is functionally essential and partially responsible for the geroprotective potential of spermidine^3^.

Two parallel studies indicated that dietary spermidine supplementation can enhance eEF5/eIF5A hypusination, cerebral mitochondrial function and cognition in aging *Drosophila melanogaster* (*D. melanogaster*) and mice^31,101^. In the *D. melanogaster* brain, hypusinated eIF5A levels decline with age but can be boosted by dietary spermidine, which serves as the amino-butyl group donor to boost the synthesis of hypusine (N^ε^-[4-amino-2-hydroxybutyl]-lysine) at a specific lysine residue of eIF5A^101^.

Dietary spermidine can pass through the blood-brain barrier (BBB) in mice and enhances hippocampal eIF5A hypusination and mitochondrial function, while spermidine feeding in aged mice changes animal behavior in homecage environment tasks, improves spatial learning and increases hippocampal respiratory capacity^31^.

Both spermidine level and eIF5A hypusination tend to decline with aging in vivo, but this decline appears to be reversable through dietary spermidine supplementation^31,101^. The exhaustion of spermidine and reduction of eIF5A hypusination likely result from decreased activity of ODC1, a rate-limiting step in polyamine biosynthesis. In fact, the activity of ODC1 declines in several tissue types, such as the liver and heart of rats, while increased activity of spermidine/spermine-*N*1-acetyltransferase (SAT1) suggests enhanced catabolism with chronological aging^99,102,103^. These findings suggest potential utility of spermidine for protection against age-related organ dysfunction.

Prior to disease onset, reduced eIF5A hypusination can be observed in axons of cultured neurons as well as adult sciatic nerves of mutant fused in sarcoma (FUS) mice, which develop familial amyotrophic lateral sclerosis (ALS)^104^. Conversely, spermidine treatment rescues local translational decreases caused by reduced hypusination, and alleviates FUS-mediated impairments such as a weakened axonal network and disrupted neuronal activity.

A study investigating the contribution of the hypusination pathway to gastric inflammation induced by *Helicobacter pylori* (*H. pylori*) suggests that epithelial hypusination promotes *H. pylori*-driven gastric inflammation without affecting bacterial persistence, while targeting DHPS-dependent eIF5A hypusination may represent a novel therapeutic solution to limit *H. pylori*-associated mucosal injury as well as disease progression^105^. Spermidine is the substrate for DHPS, which catalyzes the conjugation of the amino acid hypusine to EIF5A, while hypusinated EIF5A (EIF5A^Hyp^) can bind specific mRNAs and initiate the translation^106^. Another recent study reported that DHPS is required for the differentiation and maintenance of tissue-resident macrophages (RTM)^107^. DHPS-deficient macrophages are also defective in critical homeostatic RTM functions such as efferocytosis and tissue maintenance, highlighting a cell-intrinsic and tissue-agnostic pathway that mediates differentiation of monocyte-derived macrophages into RTMs.

## The influence of spermidine on age-related hallmarks

### Genomic instability

During organismal aging, DNA damage accumulates despite sophisticated repair mechanisms, but whether polyamine supplementation changes genomic stability, DNA repair capacity or mutation frequency remains underexplored. In a study to determine the antimutagenic potential of polyamines, spermidine and putrescine appeared to reduce the number of spontaneous revertants in modified Ames tests^108^. Another study evaluated the impact of spermidine on genome maintenance and DNA damage of *Medicago truncatula*, and found that the mitigating effects of spermidine decreased during seedling development, causing increased appearance of aberrant phenotypes^109^. A 6-month administration of spermidine in the drinking water to aged mice can significantly attenuate distinct age-associated phenotypes, a process associated with reduced telomere attrition, supporting a distinct mechanism underlying the anti-aging effects of spermidine^110^.

Polyamines promote the DNA strand exchange activity of RAD51 recombinase, while such an effect of polyamines on RAD51 depends on their ability to enhance the capture of homologous duplex DNA and synaptic complex formation by the RAD51-single strand DNA nucleoprotein filament, supporting a novel function of polyamines in genome integrity maintenance *via* homology-directed DNA repair^111^. Under certain conditions, polyamine may promote the interaction of copper and iron ions with DNA, resulting in the formation of ROS in close proximity to DNA, although spermine is known to protect DNA from the damage produced by ROS generated by different agents^112^. As aging is associated with various hallmarks including impaired autophagy and accumulation of senescent cells, whether spermidine promotes senescent cell clearance by enhancing autophagy or oxidative stress remains underexplored.

Polyamines serve as substrates for oxidation reactions that produce hydrogen peroxide (H_2_O_2_) both intra- and extracellularly, a source of cellular stress that causes DNA damage. Since intracellular concentrations of polyamines can reach millimolar levels, the amount of H_2_O_2_ produced *via* their catabolism, coupled with a reduction in protective polyamines, can cause sufficient oxidative damage to contribute to diverse pathologies^113^. However, polyamines such as spermidine can also generate protective effects against DNA damage induced by an oxygen-radical generating system by binding to DNA strands to exert its antioxidative property^114^. Furthermore, brain ischemia-reperfusion (IR) causes inflammatory, oxidative and DNA damage in the liver, while supplementation with polyamines such as putrescine *via* gavage can minimize liver damage because of their anti-inflammatory and antioxidant effects^115^. These findings inspire future research to further explore the mechanisms of polyamine in exerting differential effects.

### Mitochondrial dysfunction

Maintenance of mitochondrial integrity and function is essential for cellular homeostasis and long-term survival. However, mitochondrial dysfunction can accelerate age-related dysfunction, even independently from intracellular ROS production, mainly by restraining cellular energy supply, inducing inflammation and activating cellular death pathways^116^. A combination of mitochondrial dysfunction and insufficient autophagy can result in development of multiple age-related pathologies.

Age-related organ dysfunction is often accompanied by impaired mitochondrial biogenesis and dysregulation. Specifically, spermidine upregulates mitophagy subroutines and improves mitochondrial function in cell culture, flies and mice, partially depending on hypusination, autophagy and mitophagy^31,99,101^ (Fig. 2). Although these effects depend on mitophagy turnover and hence mitochondrial quality control (elimination of dysfunctional organelles), the polyamine metabolism pathway also stimulates the biosynthesis of mitochondrial proteins (structural proteins NDUFV2 and ATP5A1), increases mitochondrial respiration and maintains mitochondrial function^117^.

A recent study indicated that spermidine administration protected mice from CCl_4_-induced liver fibrosis by conferring on macrophages a persistent anti-inflammatory and pro-resolving function, which is characterized by increased matrix metalloproteinase (MMP) expression and enhanced mitochondrial function^118^. This finding suggests an anti-fibrotic function of spermidine attributable to its action on macrophages and opens a new avenue for treating fibrotic disorders by targeting macrophage plasticity *via* spermidine-induced mitochondrion-involved metabolic reprogramming^118^.

Spermidine treatment can increase adenosine triphosphate (ATP) production and enhance mitochondrial membrane potential (MMP), a process accompanied by reduced mitochondrial ROS levels in both young and aged human-induced pluripotent stem cell-derived neurons^119^. In the case of AD, abnormal tau affects mitochondrial function, impairing mitochondrial respiration and mitophagy. However, spermidine improves mitochondrial respiration, enhances MMP as well as ATP production in both control and mutant tau-expressing cells^120^.

### Cellular senescence

Cellular senescence occurs in response to various triggers, including DNA damage and telomere shortening, and is characterized by essentially irreversible cell cycle arrest. Senescent cells can impact both local and systemic microenvironments *via* the senescence-associated secretory phenotype (SASP)^121^. A study focusing on diabetic periodontitis reported that spermidine inhibited human periodontal ligament stem cell senescence and oxidative stress, enhanced mitochondrial function, reduced the opening of membrane permeability transition pores, and decreased STING-TBK1 phosphorylation by scavenging mitochondrial-derived dsDNA in a mitophagy-dependent manner, whereas its therapeutic effect was counteracted by incorporation of cGAMP, a STING activator^122^.

Another study aimed to determine the protective effect of spermidine in a D-galactose-induced senescence model and naturally aged rats and indicated that spermidine inhibits the age-induced increase in ROS, lipid peroxidation and protein oxidation^123^. Supplementation with exogenous spermine or genetically deficient SMOX markedly enhanced autophagy, reduced senescence and attenuated fibrosis in mouse kidneys^124^. These findings indicate that the SMOX/spermine axis may be a potentially novel therapeutic target for preventing renal fibrosis, through coordinating autophagy and antagonizing cellular senescence^124^. Moreover, age-related SMOX upregulation and accumulation of its toxic byproduct acrolein can promote oxidative damage and cellular senescence, while restraining SMOX activity or polyamine degradation attenuates senescence markers and alleviates DNA damage, indicating the value of spermine catabolism as a therapeutic target^17^.

Spermidine has the potential to modulate both mitochondrial function and proteostasis, while its supplementation can improve the responsiveness to programmed cell death-1 blockade in aged mice^125^. In yeast, prolonged overexpression of Pep4p extends chronological lifespan through its anti-necrotic function, which triggers histone hypoacetylation depending on polyamine biosynthesis and *via* enhanced intracellular levels of putrescine, spermidine and its precursor S-adenosyl-methionine (SAM)^126^. A study examining the pairwise combination of two aging-delaying plant extracts or a combination of one of these plant extracts and spermidine (or resveratrol) suggested that only pairwise combinations of naturally-occurring chemical compounds that slow aging *via* different edges, modules and nodes of the network can delay aging in a synergistic manner^127^.

Intracellular polyamine contents decrease in replicative senescent endothelial cells, but can be recovered by spermidine supplementation^128^. In plants, the expression of SAM decarboxylase and spermidine synthase gene family members can remarkably change during leaf senescence^129^. Moreover, expression patterns of *eIF5A* isoforms and genes that encode factors responsible for hypusination can differ between induced and developmental leaf senescence, while spermidine controls the onset of a different type of senescence in both eIF5A-dependent and independent manners^129^. In primary human umbilical cord mesenchymal stem cells, early spermidine intervention delays the replicative senescence and restrains premature senescence induced by H_2_O_2_ *via* SIRT3-mediated antioxidation^130^.

### Altered intercellular communication

As one of the major hallmarks of aging, dysregulated intercellular communication can result in disturbance in nutrient sensing, aberrant hormonal regulation, chronic inflammation and impaired immunosurveillance. Spermidine has anti-inflammatory activities in several mouse models, including mice developing obesity and those manifesting autoimmune diseases^2,131,132^.

Spermidine supplementation in human T cells from old donors, whose spermidine biosynthesis is remarkably inhibited, restores their autophagic capacity sufficiently to approach that of young donors’ cells^11^. Upon targeting of autophagic pathways by exogenous administration of polyamines such as spermidine or spermine, the selective AKT1 inhibitor MK-2206, or the BECN1-stabilizing anthelmintic drug niclosamide, can downregulate SARS-CoV-2 propagation in vitro in human lung cells^133^.

Spermidine enhances anticancer immunosurveillance through effects on both cancer and T cells in immunocompetent mice^134^. However, polyamines may also play a role in regulating the anticancer immune response, likely contributing to the formation of immunologically “cold” tumors that do not respond to immune checkpoint blockade^135^. In this case, polyamine metabolism, gut microbiota and the diet are interconnected to establish a tumor microenvironment that favors cancer initiation and progression.

Spermidine can regulate inflammation through inducing the formation of anti-inflammatory (M2) macrophages, a process mediated by mitochondrial reactive oxygen species (mtROS)^136^. Further study indicated that spermidine elicits an anti-inflammatory program, which is driven by mtROS-dependent AMPK activation, HIF-1α stabilization and autophagy induction in macrophages^136^. As decreased insulin/IGF signaling (IIS) extends lifespan in multiple organisms, glycine N-methyltransferase and spermidine have been reported with the potential to serve as tissue-specific modulators of IIS-mediated longevity and may help develop future therapeutics to delay aging and intervene in age-related disorders^137^.

## Spermidine in clinical trials to intervene in human diseases

It is beginning to become accepted that aging is modifiable and targetable by intervening in certain mechanisms, such as nutrient sensing, cellular senescence and gut microbiome, and it may be feasible to control these aging processes sufficiently to alleviate age-related functional decline^138^. Given the diverse implications across aging-related processes and its natural occurrence, spermidine could be a good candidate for moving toward clinical studies.

Treatment of the general aging population with compounds that may modulate age-related processes, termed geroprotectors, holds the potential to provide many benefits, including a reduction of age-related pathologies. A study querying the DrugAge database, which comprises hundreds of known geroprotectors, and cross-referencing it with a recently archived repository of compound side-effect profiling, suggested the association of 124 geroprotectors with 800 unique side-effects^139^. Surprisingly, the possibility of spermidine (together with D-glucosamine) in causing adverse effects was considered to be low, consistent with recent studies reporting the safety of low-level spermidine supplementation^5,140^. In healthy older men, high-purity spermidine (hpSPD) trihydrochloride supplementat 40 mg/day for up to 28 days is safe and well-tolerated, providing important evidence supporting the safety of hpSPD^141^. Of note, spermidine is among the more promising agents that might, after further preclinical and clinical testing, turn out to impact mammalian aging^138^.

However, in some cases, there are adverse effects associated with spermidine supplementation. The correlation between serum spermidine levels and risks of stroke was found to be consistent and reproducible in cross-sectional studies involving individuals with higher risks of stroke and those with lower risks^142^. A study examining the effect of spermidine on mechanical contractions and calcium channel current indicated that spermidine inhibits gastric smooth muscle spontaneous contraction in a concentration-dependent manner *via* inhibition of the voltage-dependent calcium current (VDCC_L_) and Ca^2+^ releasing mechanism^143^.

Despite the adverse effects reported by certain studies, recent advances and expanding literature about spermidine regarding its potential as a potential gerotherapeutic has encouraged design and initiation of clinical trials. Aged adults treated with a wheat germ extract containing concentrated polyamines suggested a subtle improvement of memory performance in a 3-month randomized, placebo-controlled and double-blind Phase IIa pilot trial^55^. While this possible beneficial effect may have been mediated by stimulation of neuromodulatory pathways in the memory system, a follow-up Phase IIb trial is essential to determine the therapeutic efficacy of spermidine supplementation and to disclose underlying neurophysiological mechanisms^55^.

Another study involving spermidine supplementation reported the safety and tolerability of a spermidine-rich plant extract in mice and older adults, suggesting the feasibility of longer-term intervention studies in human subjects to determine the impact of spermidine administration on cognition and brain integrity^5^. In light of this, a 12-month single center, randomized, double-blind and placebo-controlled phase IIb trial was conducted to investigate the impact of spermidine supplementation on memory performance in older individuals (60-90 years old) with subjective cognitive decline (SCD)^144^. However, the outputs suggest that longer-term spermidine supplementation in participants with SCD did not improve memory and biomarkers as compared with placebo, although exploratory analyses indicated possible beneficial effects on verbal memory and tissue inflammation that remain to be tested in future studies involving treatments with higher dosages^58^.

A multicentric double-blind preliminary study focusing on the effect of oral spermidine supplementation on cognitive performance of older adults (85 subjects, aged between 60 and 96 years) indicated a correlation between intake of spermidine and improved cognitive performance in the subgroup treated with a higher dose of spermidine^57^. Generally, in line with accumulating preclinical data, this study suggests that spermidine may be useful for preventing or treating age-associated cognitive decline.

Besides neurodegenerative disorders, other human pathologies have also been investigated regarding potential benefits of spermidine. A single-center, randomized, double-blind, placebo-controlled and superiority trial has been initiated, which recruited 187 patients (aged ≥ 65 years) with coronary artery disease (CAD, prior coronary artery revascularisation or myocardial infarction ≥ 3 months before screening) and preserved left ventricular ejection^145^. This is to our knowledge the first trial to assess the cardiovascular and metabolic benefit of high-dose spermidine (24 mg/day) treatment in patients with CAD, and has the potential to confer important insights into the therapeutic utility of spermidine.

Despite accumulating data regarding the cardioprotective effects of spermidine in preclinical models and the positive correlation of dietary intake with reduced cardiovascular risks, a recent Chinese study suggested a close relationship between higher serum spermidine levels and increased risk of stroke^142^. However, serum spermidine levels are positively associated with improved prognosis in individuals with acute myocardial infarction (AMI), although the underlying mechanism possibly mediated by oxidative stress has not yet been found^146^.

Chronic kidney disease (CKD) is a life-threatening condition, with constipation being a risk factor for progression. In a phase 2, randomized, double-blind and placebo-controlled trial, 150 patients with stage IIIb-IV CKD received lubiprostone (8 or 16 µg) or placebo for 24 weeks^147^. Data from Multiomics analysis suggested that lubiprostone modulated the gut microbial agmatine pathway and elevated spermidine levels, with improvement in renal mitochondrial function.

A recent study examining the effects of spermidine from Miricell™ rice germ extract recruited healthy subjects who were randomly assigned to receive 1.5 mg or 3.3 mg of spermidine daily, The study indicated that 3.3 mg/day of spermidine from Miricell® rice germ extract improved biomarkers of autophagy, neuroprotection and cardiometabolic health^148^. However, subsequent follow-up studies are needed to confirm these findings.

Depression is a stress-associated disorder and is a major global health issue. A study identified imbalanced polyamine levels and dysregulated autophagy as components of depression, and indicated that conventional antidepressant pharmacotherapy augments endogenous plasma concentrations of spermidine in patients who respond to the intervention^149^. The findings suggested that spermidine supplementation may ameliorate polyamine dysregulation and stimulate autophagy in mental stress conditions, suggesting a novel, effective and potentially well-tolerated approach for treating stress-related depressive disorders^149^.

Polyamines are functionally essential for maintaining cancer cell proliferation, a property that can be exploited to restrain tumor progression. The polyamine analogue PG-11047 is a nonfunctional competitor of spermine and has demonstrated anticancer potential in cell lines and animal models of diverse cancer types^150^. A recent study suggest that PG-11047 can be safely administered to patients with advanced refractory metastatic solid tumors or lymphoma upon combination with typical cancer drugs including bevacizumab, cisplatin, erlotinib and 5-FU on a once/week dosing schedule with therapeutic benefit^151^. N(1),N(11)-diethylnorspermine (DENSPM) is a synthetic analog of spermine, can induce polyamine depletion and prevent cancer cell proliferation. Although stable disease was observed in 18% of patients with advanced hepatocellular carcinoma (HCC) receiving DENSPM treatment in a phase 1 study, further evaluation of DENSPM monotherapy for advanced HCC remains to be justified due to insufficient evidence of clinical benefit in patients evaluated^152^.

## Concluding remarks and future perspectives

Spermidine, a member of the polyamine family, is ubiquitously found in natural sources and essential for cellular proliferation and tissue homeostasis. The intracellular concentration of spermidine declines with age and is associated with physiological dysregulation and cognitive impairment. Due to its intricate interactions with cellular metabolism, there are likely multiple modes of interaction between spermidine and its intracellular targets, which may vary in a cell type-, organ-, context- and disease type-specific manner. This is consistent with aging per se, and consistent with challenging aging as an intricate and multifactorial set of processes, but the lack of a unique mode of action may constitute a technical obstacle to future targeted pharmacological development of the small molecule compounds.

**Fig. 3:**
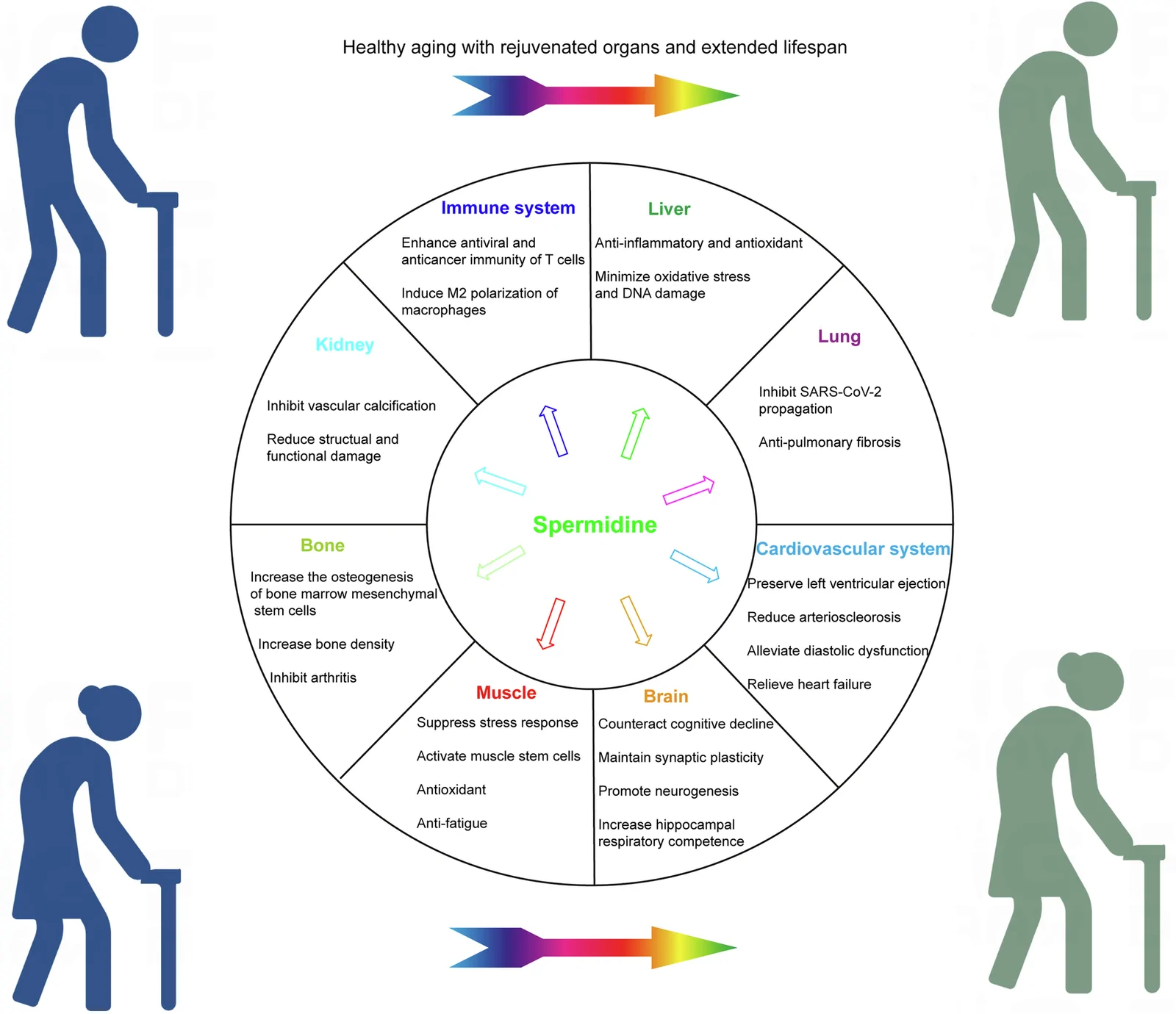
The beneficial effects that spermidine generates in cells or tissues of vital organs that degenerate with chronological aging. Generally, spermidine provokes autophagy, restrains mitochondrial damage, prevents synaptic impairment, promotes neurogenesis and neural migration, minimizes oxidative stress and dampens inflammation^3,49^. However, how spermidine supplementation prolongs healthspan and extends lifespan in model organisms remains to be further investigated. Although it is widely accepted that the geroprotective effects of spermidine involve autophagy induction, whether this can be expanded to other geroprotective mechanisms is an open, important and intriguing question that clearly warrants future research.

Recent studies have indicated that spermidine supplementation might extend lifespan and improve cognitive and cardiac function in animal models, substantiating the potential of maintaining in vivo spermidine balance for preventing and alleviating age-related dysfunction and diseases. Spermidine metabolism is precisely regulated *via* biosynthesis, degradation and transport, but age-related upregulation of SMOX and accumulation of its toxic byproduct acrolein augment oxidative damage and cause cellular senescence. Restraining SMOX activity or preventing spermidine degradation attenuates senescence markers and reduces DNA damage, lending support for spermine catabolism being a therapeutic target. Moreover, spermidine-dependent hypusination of eIF5A maintains protein synthesis in senescent cells. These multifaceted actions suggest that spermidine metabolism integrates redox control, translational regulation and autophagy to determine cellular activity, organ function and organismal healthspan (Fig. 3).

Despite the beneficial roles of spermidine in vivo, caution should be exercised about long-term spermidine supplementation. Clinical evaluation of spermidine must go hand in hand with multiple and rigorous safety studies. A systematic appraisal of spermidine as a biomarker in the aforementioned health conditions, in combination with metabolic flux analyses of relevant pathways, may help distinguish physiological and pathological changes in aging and age-related chronic diseases and could improve our understanding of human pathogenesis and disease progression. In this regard, disturbed balances in the polyamine pathway contributing to natural or progeroid aging should be dissected prior to medical intervention. To eliminate inherent or therapy-induced senescent cells, senotherapeutics have gained increasing attention in recent years. A number of naturally derived agents such as rapamycin^153^, quercetin^154^, piperlongumine^155^, fisetin^156^, luteolin^157^ and procyanidin C1^158^, have proved to be excellent senolytics or senomorphics. How spermidine compares to these natural compounds, whether it has senotherapeutic activities and what its main targets are in senescent cells merit rigorous investigation. Spermidine can mitigate bleomycin-induced premature cellular senescence, enhance beclin-1-dependent autophagy and autophagy modulators in idiopathic pulmonary fibrosis (IPF) fibroblasts and bleomycin-induced mouse lungs^159^. However, further details including the molecular targets of spermidine in these cases remain yet unclear.

Spermidine affects most of the hallmarks of aging. Fortunately, spermidine appears to be safe for humans, and the bioavailability of spermidine is reasonably good in mammals. Although animal studies evidently support geroprotective effects of spermidine, human clinical data remain limited, with variable outcomes likely attributable to drug bioavailability, differences in dosing regimens and metabolic conversion (Table 1). Prospective efforts combining dietary or probiotic spermidine enhancement and personalized metabolic interventions hold promise for extension of healthspan. Together, maintaining optimal spermidine levels has emerged as a novel and possibly important approach to counteract age-related dysfunction and alleviate age-related conditions. In clinical trials, optimal dosing and scheduling, and the effects of externally supplied spermidine on cellular, intestinal and systemic polyamine metabolism, remain to be further determined. We anticipate that research into these critical issues will improve the design and optimize the implementation of future clinical trials to evaluate the potential geroprotective properties of spermidine.

**Table 1:** Clinical trials involving spermidine administration to intervene against age-related dysfunction

Clinical trial no.	Project title	Subject condition	Study type and clinical phase	Status
NCT04405388	Spermidine anti-hypertension study	Arterial hypertension	Interventional; phase 3	Unknown
NCT03378843	Spermidine intake and all-cause mortality	All-cause mortality	Observational	Completed
NCT04138134	Autophagy and venous endothelial function	Venous disease	Observational	Unknown
NCT04823806	An exploratory clinical study on autophagy and multi-level molecular profiling during spermidine supplementation	Depression	Interventional	Unknown
NCT06792916	This study evaluates the clinical effect of adding spermidine gel to minimally invasive non-surgical periodontal therapy (minst) to improve periodontal health	Periodontitis	Interventional；phase 4	Recruiting
NCT07202403	Iterative design trial to assess dietary supplements and other aging-targeted therapies	Aging, longevity	Interventional; not applicable	Recruiting
NCT06792916	This study evaluates the clinical effect of adding spermidine gel to minimally invasive non-surgical periodontal therapy (minst) to improve periodontal health	Peridontitis	Interventional; phase 4	Recruiting
NCT07058974	A study of exercise and pharmacologic intervention on systemic inflammation	Systemic inflammation in smokers	Interventional；phase 1	Recruiting
NCT06445569	Safety and efficacy of kh-1 for stimulating autophagy in non-diabetic adults with elevated blood glucose concentration	Prediabetes	Interventional; not applicable	Completed
NCT07383311	Autophagy-enhancers to reduce sleep disturbances	Mild cognitive impairment due to Alzheimer’s disease	Interventional; not applicable	Recruiting
NCT06186102	Polyamine treatment in elderly patients with coronary artery disease	Cardiovascular diseases, cognition disorder, cognitive impairment, diabetes mellitus, type 2	Interventional；phase 2	Active, not recruiting
NCT03094546	Polyamine-enriched diet in elderly individuals with subjective cognitive decline (SmartAge)	Subjective cognitive decline	Interventional；phase 2	Completed
NCT05421546	Improving vaccination in older adults by inducing autophagy with spermidine	Older people (people aged greater than 65 years old) that received coronavirus vaccine	Interventional; phase not applicable	Completed

## Data Availability

All data employed in this study are essentially from public literatures or federal databases, and openly available to the scientific community.
